# Population pharmacokinetics of piperacillin-tazobactam in the plasma and cerebrospinal fluid of critically ill patients

**DOI:** 10.1128/aac.00601-24

**Published:** 2024-12-19

**Authors:** Nilesh Kumta, Aaron J. Heffernan, Menino Osbert Cotta, Xin Liu, Suzanne Parker, Steven Wallis, Amelia Livermore, Therese Starr, Wai Tat Wong, Gavin M. Joynt, Jeffrey Lipman, Jason A. Roberts

**Affiliations:** 1 The University of Queensland Centre for Clinical Research, Faculty of Medicine, University of Queensland 303224 , Herston, Brisbane, Queensland, Australia; 2 School of Medicine, Griffith University 63617 , Southport, Queensland, Australia; 3 Department of Intensive Care Medicine, Royal Brisbane and Women's Hospital 550021 , Herston, Brisbane, Queensland, Australia; 4 Department of Anaesthesia and Intensive Care, The Chinese University of Hong Kong 71024 , Hong Kong, China; 5 Division of Anaesthesiology Critical Care Emergency and Pain Medicine, Nimes University Hospital, University of Montpellier 149920 , Nimes, France; 6 Jamieson Trauma Institute, Royal Brisbane and Women's Hospital 3883 , Herston, Brisbane, Queensland, Australia; 7 Pharmacy Department, Royal Brisbane and Women's Hospital 3883 , Herston, Brisbane, Queensland, Australia; University of Pittsburgh School of Medicine, Pittsburgh, Pennsylvania, USA

**Keywords:** ventriculitis, ventriculostomy-associated infection, antibiotics, pharmacokinetic/pharmacodynamic, piperacillin-tazobactam, cerebrospinal fluid, critical illness

## Abstract

Ventriculitis in neurocritical care patients leads to significant morbidity and mortality. Antibiotic dose optimization targeting pharmacokinetic/pharmacodynamic (PK/PD) exposures associated with improved bacterial killing may improve therapeutic outcomes. We sought to develop and apply a population PK model in infected critically ill patients to determine optimal piperacillin-tazobactam (PTZ) dosing regimens to achieve target cerebrospinal fluid (CSF) exposures. Neurosurgical patients with external ventricular drains and receiving PTZ treatment were recruited and had plasma and CSF samples collected and assayed. A population PK model was developed using plasma and CSF piperacillin and tazobactam concentrations. Eight patients were recruited. Median age was 59 years, median weight was 70 kg, and five patients were female. The median creatinine clearance was 84 mL/min/1.73 m^2^ (range 52–163). Substantial inter-individual PK variability was apparent, particularly in CSF. Piperacillin penetration into CSF had a median of 3.73% (range 0.73%–7.66%), and tazobactam CSF penetration was not predictable. Dosing recommendations to optimize CSF exposures for the treatment of ventriculitis were not possible due to substantial PK variability and very low drug penetration. High plasma PTZ exposures may not translate to effective exposures in CSF.

## INTRODUCTION

Nosocomial infections in the intensive care unit (ICU) are a major source of morbidity and mortality. In neurocritical care patients, surgical insertion of external ventricular drains (EVD) may be associated with ventriculitis or ventriculostomy-associated infection (VAI) (1). Antibiotic dose optimization that maximizes pharmacokinetic/pharmacodynamic (PK/PD) exposure in the cerebrospinal fluid (CSF) and the site of infection may improve clinical outcomes in patients with VAI, potentially suppressing the emergence of antimicrobial resistance (2, 3).

Piperacillin-tazobactam (PTZ) is a penicillin and beta-lactamase inhibitor combination that is prescribed for empiric sepsis treatment due to respiratory or intra-abdominal infections. Like all beta-lactams, piperacillin is a hydrophilic molecule with a low volume of distribution (V_d_) and is predominantly cleared via renal elimination (2). Piperacillin exhibits time-dependent bactericidal activity; the minimum PK/PD target associated with improved clinical outcomes is a percentage of dosing interval that unbound drug concentration remains above pathogen minimum inhibitory concentration (MIC; %*f*T_>MIC_) of 40%–70% (2, 4–7). Critically ill patients may benefit from higher than standard exposures, with a ratio of minimum unbound concentration to MIC greater than one (*f*C_min_/MIC > 1) (4–6, 8, 9). For tazobactam, an unbound drug concentration greater than 2 mg/L for 100% of the dosing interval (100% *f*T_>2 mg/L_) is associated with increased bactericidal activity in preclinical models for Enterobacterales (10).

Neurocritical care patients with VAI frequently face antibiotic dosing-related challenges. The potentially low meningeal inflammation associated with VAI may increase the likelihood of subtherapeutic concentrations in the CSF (11). Furthermore, augmented renal clearance (ARC, creatinine clearance of ≥130 mL/min/1.73 m^2^), particularly in patients with head trauma, can increase the clearance of hydrophilic drugs such as beta-lactams (12). This variability is important to consider during dose selection for treatment of VAI, with PTZ not commonly considered a treatment option for VAI. Previous PK papers only highlight plasma exposures of PTZ or CSF exposures during treatment of meningitis. Only one previous study has described PTZ PK in CSF among patients with low meningeal inflammation. This study, however, did not provide a robust description of PK variability or quantification of PK/PD exposures in CSF (13, 14).

The aims of this study were to (i) describe the plasma and CSF population PK of piperacillin and tazobactam in infected critically ill patients with an EVD *in situ* and (ii) apply the final PK model for dosing simulations to determine the optimal PTZ dosing regimens required to achieve PK/PD targets for piperacillin and tazobactam in CSF and plasma.

## MATERIALS AND METHODS

### Patient population

This study was performed at two university-associated tertiary-level ICUs. Patients were included in the study if they met the following inclusion criteria: (i) age 18–85 years, (ii) EVD *in situ*, (iii) receiving PTZ treatment, and (iv) a diagnosis of VAI or extracranial infection. Notably, definitions for VAI are not uniform, with some studies using positive CSF culture, others positive CSF culture along with clinical or microbiological criteria, and others using either positive culture or clinical or microbiological criteria to diagnose VAI (1). In this study, VAI was diagnosed by the treating intensivist. Extracranial infections were considered acceptable because, like VAI, they are not known to affect meningeal permeability and therefore are likely to provide similar CSF penetration as seen in VAI. Exclusion criteria were as follows: (i) small ventricles on computed tomography with intracranial pressure >20 mmHg deemed by the treating intensivist, (ii) presence of renal impairment defined as the need for replacement therapy or a plasma creatinine concentration >200 µmol/L, (iii) pre-existing hepatic dysfunction defined as gamma-glutamyl transferase >200 IU/L, (iv) allergy to beta-lactams, (v) pregnancy, and (vi) patients in whom death was deemed inevitable within 24 h. Patient characteristics such as age (years), weight (kg), height (cm), body mass index, suspected infection source, clinical morality prediction scores (including Sequential Organ Failure Assessment [SOFA] and Acute Physiology and Chronic Health Evaluation [APACHE] scores), microbiological results (if available), serum albumin (g/L), CSF parameters (including pleocytosis, glycorrhachia [mmol/L], red blood cell count, and protein [g/L]), serum creatinine (µmol/L), creatinine clearance (mL/min, calculated per Cockcroft-Gault equation), and presence of ARC were prospectively recorded.

### Sample times, handling, storage, and measurement

Blood sampling was obtained 0.5, 1, 1.5, 2, 4, and 6 h after infusion commencement, and CSF samples were obtained 0.5, 2, 4, and 6 h after the start of infusion for patients receiving an intermittent bolus regimen. For one patient who received a continuous infusion, blood and CSF samples were obtained 17–26 h after infusion commencement. Blood samples were immediately stored in an ice bath and centrifuged within 6 h, and 1.5–1.8 mL of the ensuing plasma was transferred to a labeled cryovial which was immediately stored at −20°C. CSF samples were immediately placed on ice and centrifuged within 60 minutes. Samples were frozen at −20°C within 4 h and then stored at −80°C within 2 weeks. Urine was also collected over the sampling period and stored alongside the plasma specimen. All samples were subsequently transferred with dry ice to the University of Queensland Centre for Clinical Research, Brisbane.

Total concentrations of piperacillin and tazobactam were measured in plasma, CSF, and urine by a validated ultra-high performance liquid chromatography-mass spectrometry (UHPLC-MS/MS) method on a Nexera UHPLC connected to an 8030+ triple quadrupole mass spectrometer (Shimadzu, Kyoto, Japan). Samples were assayed in batches alongside calibrators, and quality controls and results were subject to batch acceptance criteria (US FDA, Guidance for Industry: Bioanalytical Method Validation, May 2018) (15).

Cerebrospinal fluid samples were processed as plasma samples. Plasma and CSF are biological liquids with similar potential interferences in terms of salts and proteins, and a stable isotope-labeled PTZ internal standard was used that is resilient against ion suppression. Samples (10 µL of plasma and CSF) were combined with deuterated piperacillin and sulbactam internal standard, and acetonitrile (40 ul) was added to precipitate any proteins. An aliquot of the solution was injected into the UHPLC-MS/MS. Chromatographic separation was achieved via a Shim-pack analytical guard column as the stationary phase.

Piperacillin was monitored by a positive mode electrospray optimized with multiple reaction monitoring at fragmentation ions of 518.00→143.00. Deuterated piperacillin was monitored in positive mode at 523.00→148.00. Tazobactam and sulbactam were monitored by negative mode electrospray with multiple reaction monitoring at fragmentation ions of 299.20→138.00 and 232.35→139.90, respectively. Precision was within 5.8%, and accuracy was within 10.0% at the tested plasma quality control piperacillin concentrations of 1.5, 50, and 400 mg/L. For tazobactam, precision was within 7.0%, and accuracy was within 7.6% at the tested plasma quality control concentrations of 1.875, 6.25, and 50.0 mg/L. The lower limits of quantification for piperacillin and tazobactam in both plasma and CSF were 0.5 mg/L and 0.625 mg/L, respectively.

In the setting of meningitis, concentrations of inflammatory markers such as interleukin-1 (IL-1), IL-6, and procalcitonin in CSF are often elevated (16–18). Thus, to quantify the degree of meningeal inflammation in enrolled patients, IL-1 concentrations were measured in plasma and CSF samples taken either just before antibiotic dosing or after the first dose had been administered using the Abcam IL-1 Human Enzyme-Linked Immunosorbent Assay kit (Abcam, Cambridge, UK) over a calibration range of 0.412–100 pg/mL. Cerebrospinal fluid samples were processed as plasma samples following the kit instructions.

### Population pharmacokinetic model development

Population PK analyses in CSF and plasma were performed using the nonlinear mixed-effects modeling program Monolix version 2023R1 (LIXOFT, Antony, France), implementing the stochastic approximation expectation maximization algorithm. Individual estimates for PK parameters were assumed to follow a log-normal distribution. The between-subject variability (BSV) was described using an exponential model according to the equation $\theta_{j}=\theta_{p}\times exp\left(\eta_{j}\right)$, where *θ_j_* is the estimate for a PK parameter in the *j^th^* patient as predicted by the model, *θ_p_* is the typical population PK parameter value, and *η_j_* is a random variable from a normal distribution with zero mean and variance *ω*^2^, which is estimated.

For piperacillin, one- and two-compartment models were tested, with the CSF compartment subsequently integrated into the plasma model. Models with and without elimination from the CSF compartment were compared. For tazobactam, one- and two-compartment models were compared to model plasma concentrations. Cerebrospinal fluid concentrations of tazobactam were below the lower limit of quantification (BLOQ) for most samples; as such, these data were not used for population PK analysis. Censoring of the BLOQ was trialed but did not improve model fit in CSF and so was not used in the final model.

Several error models were tested for describing the residual variability (ε). Model selection was based on the accuracy of parameter estimates, visual inspection of goodness-of-fit (GOF) plots, and numerical assessment of the objective function value and corrected Bayesian information criterion. Clinical and demographic patient variables such as age, sex, weight, height, body mass index, APACHE and SOFA scores, serum albumin, creatinine clearance (calculated using the Cockcroft-Gault equation using total body weight and expressed in mL/min/1.73 m^2^), and serum alanine aminotransferase levels were considered for inclusion as covariates in the modeling process, using stepwise forward inclusion and backward elimination approaches. A reduction in the negative log-likelihood ratio (-2LL) of at least 3.84 (*P* < 0.05) and an increase of greater than 10.83 (*P* < 0.001) were required for a covariate to be considered significant for the forward inclusion and backward elimination steps, respectively.

### Model evaluation

Model evaluation was based on GOF plots, including observed vs individual and population predicted values, weighted individual residuals vs individual predictions and time, and plots of normalized prediction distribution error (NPDE) vs population predictions and time. The visual predictive check (VPC) was performed using 500 simulations with the final model. The accuracy of the final model was also examined using a bootstrap method: a 1,000-run bootstrap resampling procedure was performed in Monolix using the Rsmlx (R Speaks “Monolix,” version 2023.1.1) package in R software (version 4.3.1). The median, 2.5%, and 97.5% values obtained from the 1,000 bootstrap runs for each parameter were calculated and compared to the estimates from the original data.

### Penetration into CSF

Monolix was used to calculate piperacillin and tazobactam area under the concentration-time curve (AUC) in plasma and CSF using the individual predicted AUCs in each compartment; penetration into CSF was described by converting the CSF/plasma AUC ratio into a percentage.

## RESULTS

### Study population

From the eight patients enrolled in the study, 45 plasma and 30 CSF samples were obtained. Demographic and clinical details are presented in Table 1. Table 2 presents PTZ dosing, patient renal function, and plasma and CSF concentrations observed.

**TABLE 1:** Patient demographic and clinical details^*a*^

Characteristic	Value (*N* = 8)
Age, years, median (range)	59 (42–75)
Female sex, n (%)	5 (62.5)
Weight, kg, median (range)	70 (47–110)
Albumin, g/L, median (range)	25.5 (22–32)
Creatinine clearance, mL/min, median (range)	84 (52–163)
Augmented renal clearance^*b*^, n (%)	1 (12.5)
CSF protein, g/L, median (range)	0.73 (0.19–1.8)
CSF protein:serum albumin, median (range)	0.016 (0.006–0.056)
CSF volume drained from EVD, mL, median (range)	37.5 (0–67)
SOFA score, median (range)	7.5 (2–12)
APACHE scores, median (range)	18 (12–27)
Infection diagnosis, n (%)	
Pneumonia	7 (87.5)
Ventriculostomy-associated infection	1 (12.5)

^*a*^ CSF, cerebrospinal fluid; EVD, external ventricular drain; SOFA, sequential organ failure assessment; APACHE, acute physiology and chronic health evaluation.

^*b*^ Defined as creatinine clearance ≥ 130 mL/min.

**TABLE 2:** Piperacillin-tazobactam dosing, patient renal function, and plasma and cerebrospinal fluid concentrations achieved^*a*^

Patient	Creatinine clearance (mL/min)	PTZ dosing	Plasma sample piperacillin concentration range (mg/L)	Plasma sample tazobactam concentration range (mg/L)	CSF sample piperacillin concentration range (mg/L)	CSF sample tazobactam concentration range (mg/L)
RB04	88	4.5 g q6h	5.13–212.47	0.79–23.74	BLOQ–0.98	BLOQ
RB05	163	4.5 g q6h	6.65–157.74	1.42–19.46	0.57–2.06	BLOQ
RB10	64	4.5 g q6h	17.59–286.15	5.08–32.71	3.60–5.72	1.15–1.62
RB11	52	CI 13.1 g over sampling period	39.13–79.08	8.73–13.15	1.62–2.72	BLOQ–0.645
RB12	110	4.5 g q6h	1.87–146.98	BLOQ–16.13	2.30–2.91	BLOQ
HK06	78	4.5 g q6h	8.89–164.79	1.42–18.45	1.54–3.50	BLOQ
HK07	119	4.5 g q6h	9.50–265.92	1.52–30.33	4.48–7.47	0.79–0.86
HK08	82	4.5 g q6h	21.31–244.97	3.19–27.91	4.28–6.31	0.74–1.26

^*a*^ BLOQ, below limit of quantification; PTZ, piperacillin-tazobactam; CSF, cerebrospinal fluid; CI, continuous infusion.

Measured piperacillin and tazobactam concentrations in plasma and CSF displayed substantial intra- and inter-individual variability (Table 2). Cerebrospinal fluid piperacillin and tazobactam concentrations were particularly variable. Fourteen CSF samples had undetectable tazobactam concentrations. Observed piperacillin and tazobactam plasma and CSF concentrations are presented in Figure 2. The eight enrolled patients had IL-1 measured in 16 samples (range from undetectable to 2.18 pg/mL; interquartile range 1.47 pg/mL) with highly variable concentrations observed in both plasma and CSF. The highest CSF and plasma IL-1 concentrations were 2.18 pg/mL and 2.08 pg/mL, respectively.

### Pharmacokinetic model

**Fig 1:**
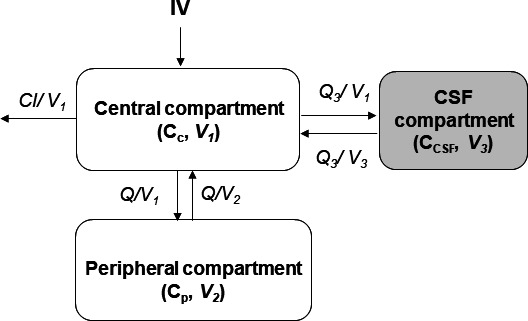
Schematic diagram of the final PK model for piperacillin after intravenous infusion. *C_c_*, plasma concentration in central compartment; *C_CSF_*, concentration in CSF; *V_1_*, volume of central compartment; *Cl*, clearance from the central compartment; *V_2_*, volume of peripheral compartment; *Q*, inter-compartment clearance between the central and peripheral compartment; *Q_3_*, inter-compartment clearance between the central and CSF compartment; *V_3_,* volume of CSF compartment.

Cerebrospinal fluid and plasma piperacillin concentrations were most adequately described by a three-compartment model without clearance from the CSF compartment (Fig. 1). Residual variability was best described by a combined (additive plus proportional) error model for plasma concentrations, while a proportional error model was selected for CSF concentrations. No covariates improved model diagnostics significantly and thus could be retained in the final model.

**Fig 2:**
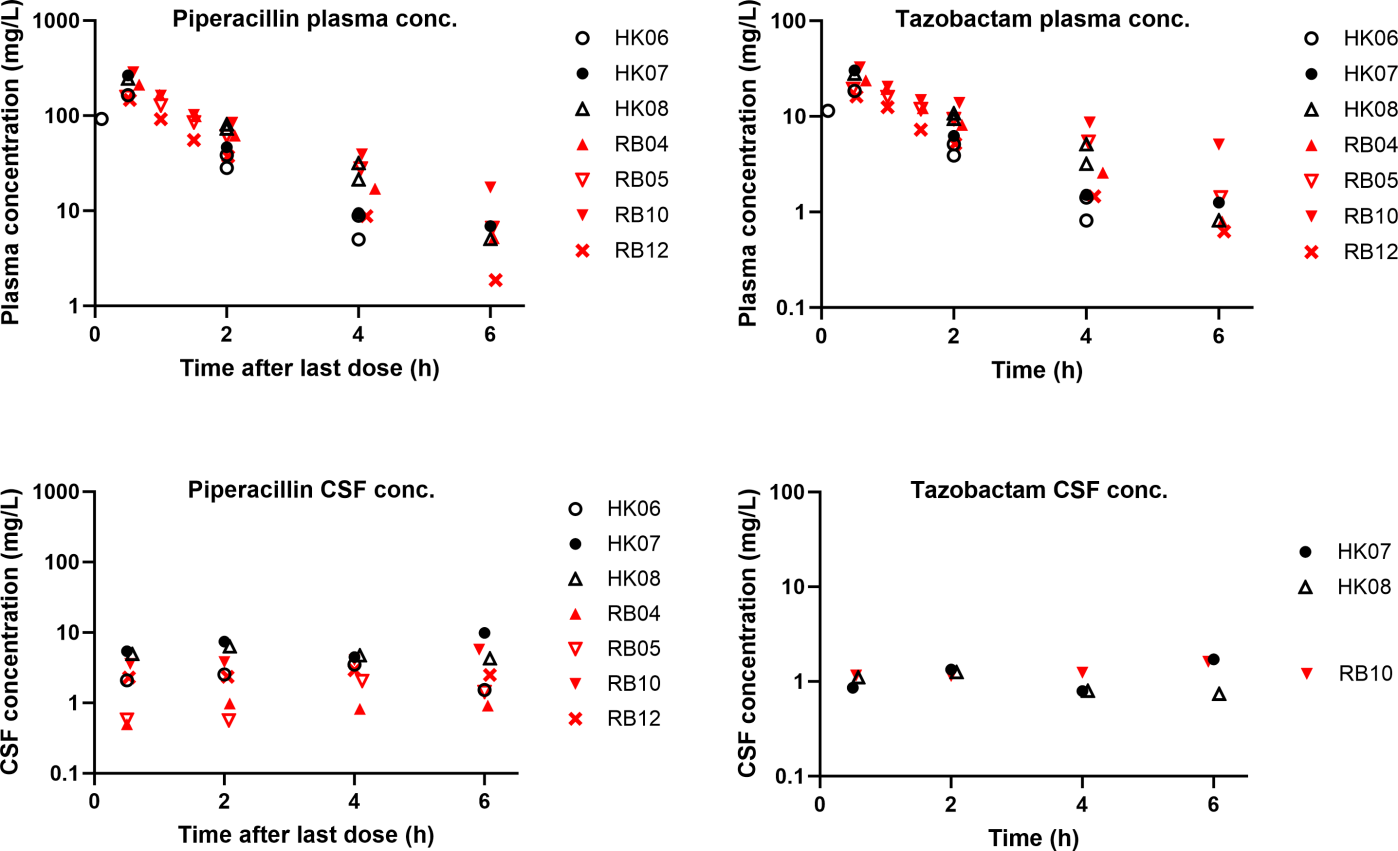
Observed plasma and CSF piperacillin and tazobactam concentrations.

For tazobactam, CSF concentrations did not show any significant changes in concentrations throughout the dosing interval making it not possible to model CSF exposures (Fig. 2). A two-compartment model with first order elimination best described the PK of tazobactam in plasma. Residual variability was best described by a combined additive and proportional error model. Likewise, no covariate effect was identified.

Fifty percent of patients did not have urine samples collected, and so modeling of urinary concentrations was not performed.

**Fig 3:**
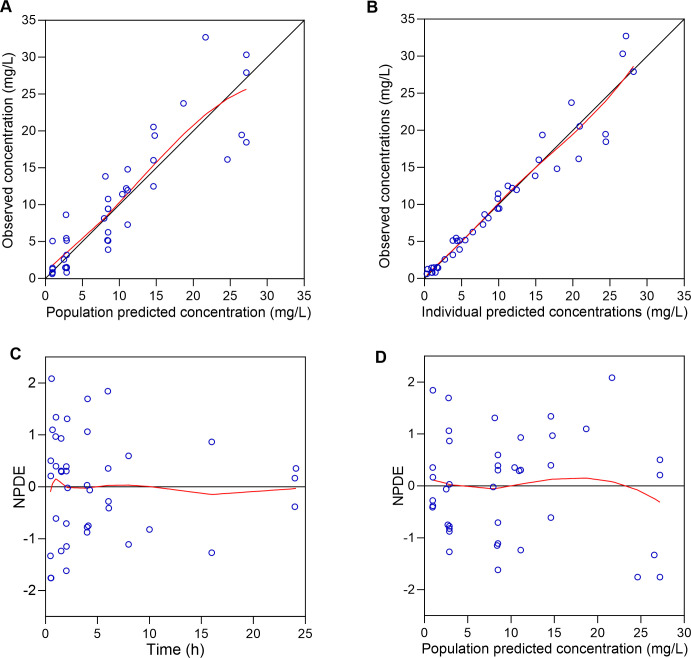
Goodness-of-fit plots for plasma piperacillin concentrations. (**A**) Observed vs population-predicted concentrations; (**B**) observed vs individual-predicted concentrations. The solid black line represents the line of identity, and the solid red line represents the spline line. (**C**) NPDE vs time; (**D**) NPDE vs population-predicted concentrations. The solid red line represents the spline line.

**Fig 4:**
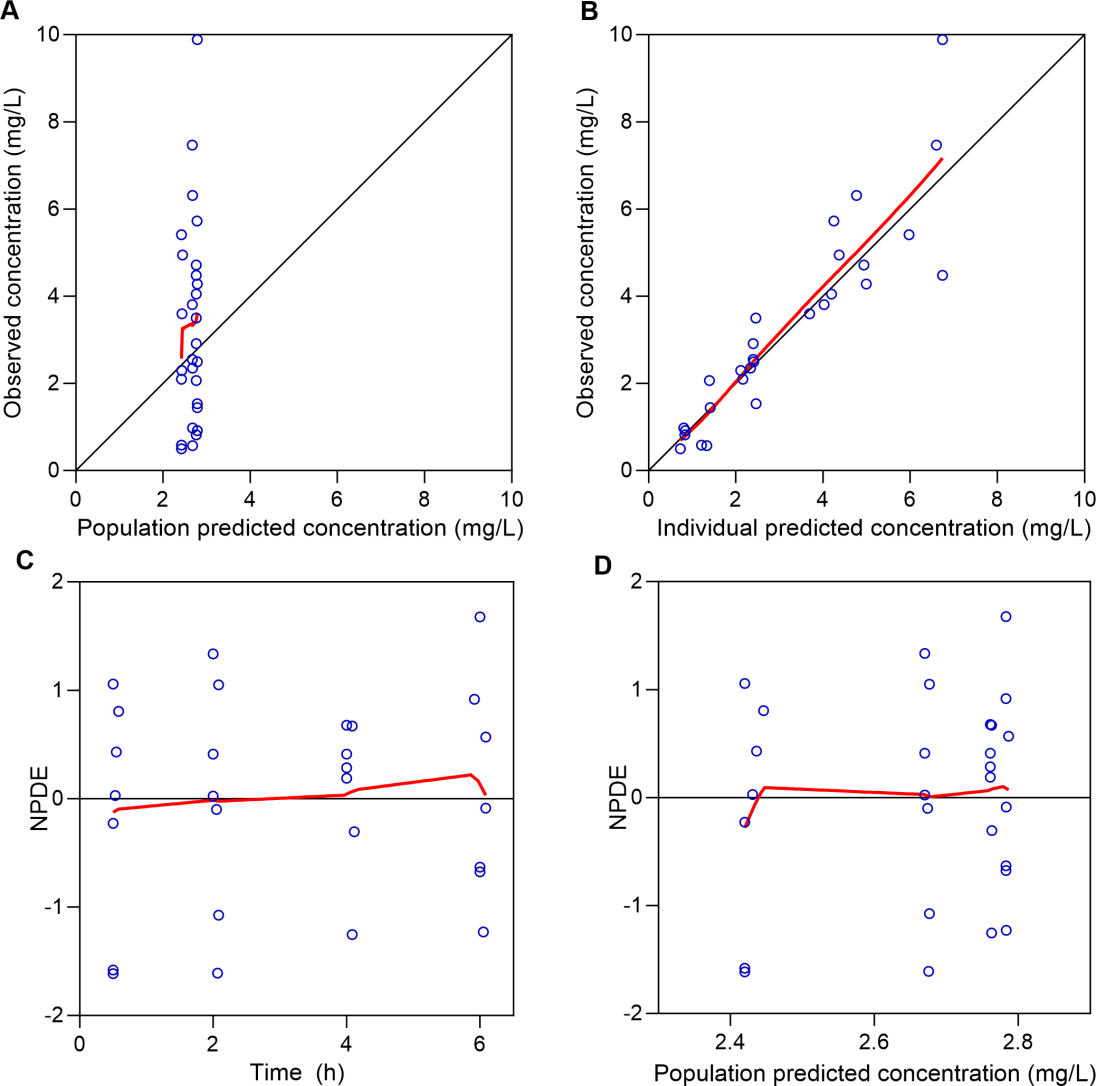
Goodness-of-fit plots for CSF piperacillin concentrations. (**A**) Observed vs population-predicted concentrations; (**B**) observed vs individual-predicted concentrations. The solid black line represents the line of identity, and the solid red line represents the spline line. (**C**) NPDE vs time; (**D**) NPDE vs population-predicted concentrations. The solid red line represents the spline line.

**Fig 5:**
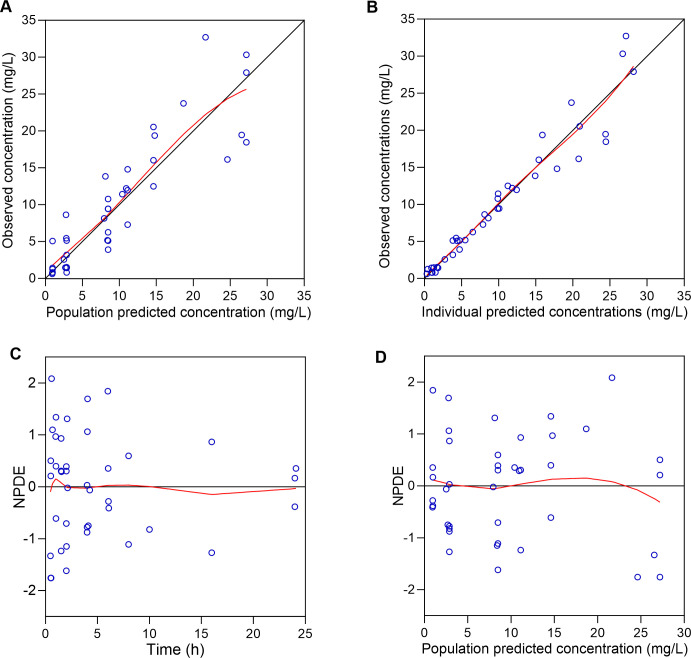
Goodness-of-fit plots for plasma tazobactam concentrations. (**A**) Observed vs population-predicted concentrations; (**B**) observed vs individual-predicted concentrations. The solid black line represents the line of identity, and the solid red line represents the spline line. (**C**) NPDE vs time; (**D**) NPDE vs population-predicted concentrations. The solid red line represents the spline line.

**Fig 6:**
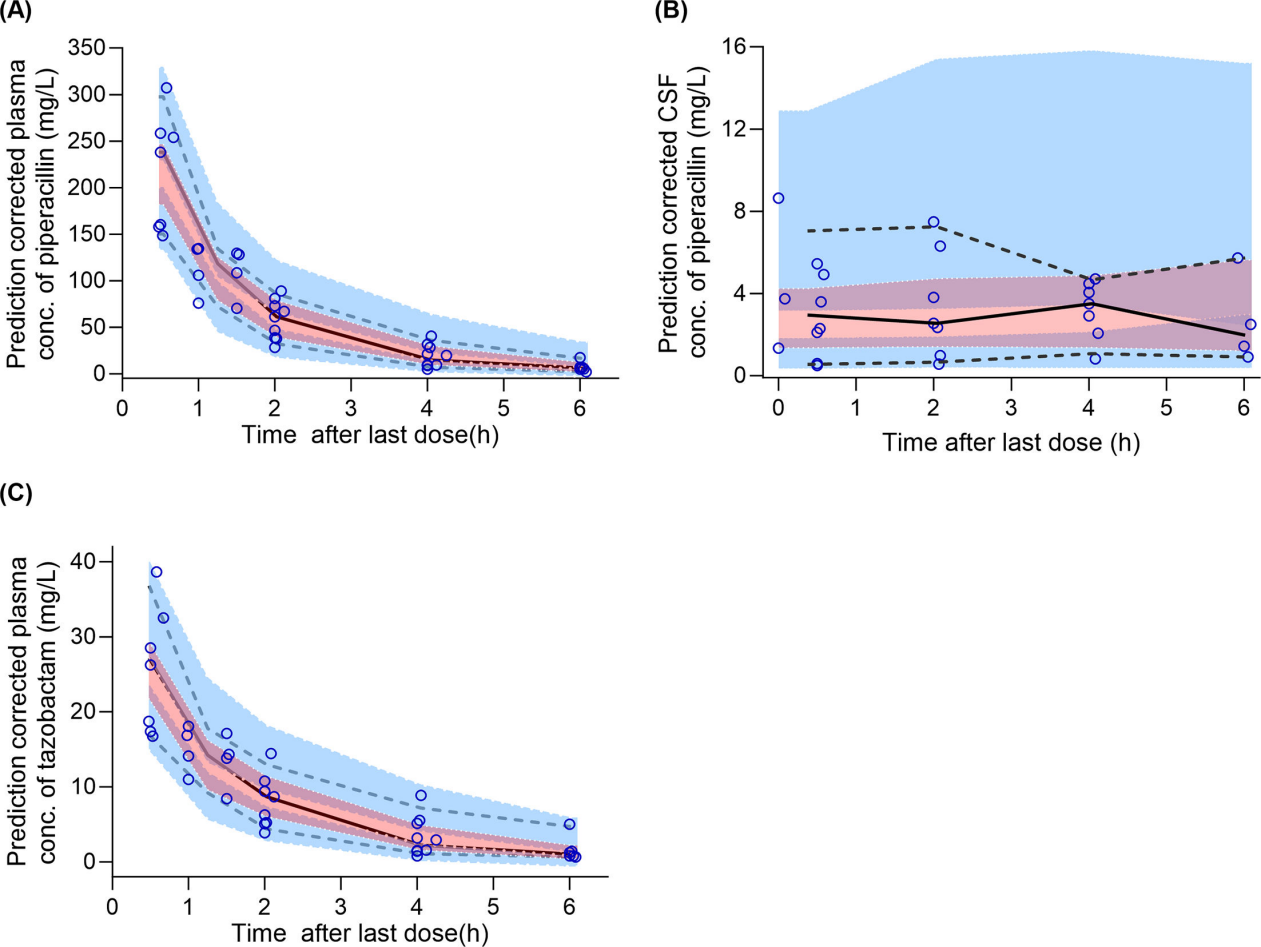
Prediction-corrected visual predictive check plots for (**A**) plasma concentrations and (**B**) CSF concentrations of piperacillin and (**C**) plasma concentrations of tazobactam. Symbols represent observations. Lines represent empirical percentiles (5th, 50th, and 95th percentile). Shaded area represents 90% prediction interval around each predicted percentile.

Pharmacokinetic parameters estimated from the final models are presented in Table 3. Figures 3 and 4 present the GOF plots for piperacillin concentrations in plasma and CSF, respectively, predicted by the model. Figure 5 presents the GOF plot for tazobactam concentrations in plasma predicted by the model. Figure 6 presents the VPC plots for the final model.

**TABLE 3:** Pharmacokinetic parameter estimates from final model^*a*^

Parameter	Piperacillin		Tazobactam	
	Estimate (%RSE)	Bootstrap median (95% CI)	Estimate (%RSE)	Bootstrap median (95% CI)
Fixed effect				
*Cl* (L/h)	12.7 (11.1)	12.6 (10.3–15.8)	11.7 (12.7)	11.4 (9.00–15.5)
*V_1_* (L)	13.4 (15.0)	13.5 (9.35–17.6)	7.64 (43.2)	7.17 (0.18–18.2)
*Q* (L/h)	7.25 (3.41)	6.85 (1.85–70.7)	46.5 (64.5)	48.8 (6.18–216)
*V_2_* (L)	4.99 (28.5)	4.98 (2.94–12.6)	12.0 (23.6)	15.7 (5.08–20.4)
*Q_3_* (L/h)	0.00024 (85.3)	0.00025 (0.00013–0.00047)		
*V_3_* (L)	0.16 (80.2)	0.16 (0.12–0.25)		
Random effect				
*BSV*_*Cl* (%)	29.5 (27.8)	27.1 (10.6–36.1)	33.6 (28.7)	33.4 (9.85–40.4)
*BSV*_ *V_1_* (%)	27.0 (36.0)	24.8 (4.25–35.8)	28.7 (67.7)	25.4 (4.41–49.2)
*BSV_ Q_3_* (%)	86.3 (28.4)	75.6 (22.7–124)		
Error model parameter				
Additive residual_plasma (mg/L)	2.10 (31.7)	2.26 (0.45–3.75)	0.47 (28.6)	0.51 (0.22–0.88)
Proportional_plasma	0.08 (31.1)	0.07 (0.01–0.12)	0.15 (20.0)	0.14 (0.08–0.18)
Proportional_CSF	0.30 (16.3)	0.30 (0.18–0.42)		

^*a*^ *V_1_*, volume of central compartment; *Cl*, clearance from the central compartment; *V_2_*, volume of peripheral compartment; *Q*, inter-compartment clearance between the central and peripheral compartment; *Q_3_*, inter-compartment clearance between the central and CSF compartment; *V_3_*, volume of CSF compartment; BSV, between-subject variability; RSE, relative standard error; CI, confidence interval.

### Penetration into cerebrospinal fluid

Piperacillin penetration into CSF was variable and low. The median (interquartile range) predicted AUC in CSF was 3.73% (0.73%–7.66%) of the predicted AUC in plasma. Median predicted trough piperacillin concentrations in plasma (6h post-dosing) were 6.67 mg/L (95% CI, 1.79–13.8 mg/L); in contrast, median CSF piperacillin concentrations were 2.46 mg/L (95% CI, 0.93–6.47 mg/L). Cerebrospinal fluid penetration of tazobactam was not able to be reliably estimated due to the large number of samples (47%) with undetectable tazobactam concentrations.

## DISCUSSION

This study has highlighted the PK variability of piperacillin and tazobactam in neurocritical care patients, particularly in CSF. This clearly complicates the attainment of effective piperacillin and tazobactam CSF exposures during the treatment of VAI. Despite this, a CSF PK model for piperacillin and plasma PK models for piperacillin and tazobactam were able to be described in this study. However, the variability and “flat” concentration-time profile precluded the development of a reliable PK model to describe the exposure in the CSF for tazobactam. The piperacillin model was also not reliable enough to perform dosing simulations of CSF exposures. The variability of PK parameters for piperacillin and tazobactam in our study was comparable to other studies in critically ill patients (19–22), which ranged from 9% to 120% in terms of percent between-subject variability (%BSV), relative standard error (%RSE), and coefficients of variation (%CV). Conversely, %CV for all parameters in a two-compartment population PK model of piperacillin was 3%–13% in healthy volunteers (23). However, a direct comparison between studies is made challenging by the different measures of dispersion used.

Piperacillin and tazobactam concentrations in CSF were appreciably lower than in plasma, with considerable variability over the dosing interval. Cerebrospinal fluid piperacillin concentrations were similar to those reported previously (<0.37–8.67 mg/L) (13). Such exposures are unlikely to be therapeutic in critically ill patients where the clinical MIC susceptibility breakpoint may be as high as 16 mg/L for infections caused by *Pseudomonas aeruginosa*. In our study, the highest CSF piperacillin concentration attained was 7.5 mg/L. This makes it unlikely that CSF concentrations of piperacillin were above pathogen MIC for a significant proportion of the dosing interval. Importantly, the limited CSF concentration data and small sample size in our study precluded the model from predicting the population PK parameters of tazobactam in CSF accurately, although the low concentrations likely mean there is no clinical role for tazobactam in VAI anyway.

The presence of variable and low meningeal inflammation in the context of VAI may, at least in part, contribute to low CSF piperacillin and tazobactam concentrations. Meningitis is characterized by reduced CSF drainage and enhanced endothelial permeability of the blood-CSF barrier in contrast to ventriculitis or extracranial infections (24, 25). Quantification of meningeal inflammation in neurocritical care patients may also be challenging, as suggested by the highly variable and frequently undetectable CSF IL-1 concentrations seen in our patients. The degree and variability of piperacillin penetration into CSF in this study were in keeping with a previous study (13), which described higher (mean CSF-to-serum AUC ratio 0.17 vs 0.05) but more variable (standard deviation 0.23 vs 0.03) tazobactam vs piperacillin CSF penetration. Low CSF penetration would be consistent with the hydrophilic nature and moderate protein binding of piperacillin (24), while tazobactam’s lower molecular weight but greater hydrophilicity and degree of ionization in body fluids would predict similar CSF penetration to piperacillin (26). Given the variable and low CSF penetration of piperacillin, high plasma exposures that may be achieved using continuous infusions may not necessarily translate to high CSF exposures. The unquantifiable CSF penetration of tazobactam in this study also makes it difficult to predict the effect of high plasma tazobactam exposures on CSF exposures. The one study patient who received a continuous infusion did not have significantly different CSF piperacillin or tazobactam concentrations compared to the study population.

The plasma PK parameters of piperacillin obtained in our study, including V_d_ and clearance, differed to those described in many studies involving critically ill patients (20, 22, 27–29), although piperacillin V_d_ was comparably small (7–14L) to some patients with critical illness (30–32) and healthy volunteers (33). However, significant inter-study variances in PK parameters of patients with critical illness are common and may be exacerbated by different modeling techniques, patient demographics, and dosing and sampling regimens. Aggressive fluid resuscitation and enhanced capillary permeability in sepsis can increase V_d_ of hydrophilic drugs such as piperacillin and tazobactam (34, 35). This may reduce concentrations in plasma (and possibly CSF), potentially necessitating higher loading doses. Interestingly, tazobactam V_d_ in our study (7.6 L) was lower than in healthy volunteers (19 L) (36), potentially due to our patients having a lower mean weight (71.5 kg) than the general population (37).

The clearance of piperacillin is highly variable among critically ill patients (20, 22, 28–30, 32, 33). Likewise, tazobactam clearance in the present study (11.7 L/h) was more than twice that described previously (5.3 L/h) in critical illness (21) but lower than in healthy volunteers (20 L/h) (36). In our study, clearance of piperacillin or tazobactam was not influenced by creatinine clearance or any other covariates—similar to another population PK study involving piperacillin (32). In contrast, previous population PK analyses have demonstrated direct correlations between creatinine clearance (27, 28) and both creatinine clearance and total body weight (30) on piperacillin clearance. Sepsis often results in multiorgan dysfunction, including acute kidney injury that may reduce the clearance of piperacillin. Contrarily, in a significant proportion of neurocritical care patients, ARC (12) can increase plasma piperacillin clearance, reducing plasma (and potentially CSF) piperacillin concentrations. Only one patient displayed ARC in the present study, which may not necessarily be representative of the broader neurocritical care population.

The small sample size and sparse convenient sampling preclude reliable model-predicted piperacillin and tazobactam CSF PK parameters, as well as an accurate assessment of the likely PK variability associated with critically ill patients. The fact that only one patient received a continuous infusion is another relevant limitation to be considered when interpreting the results. The MIC distribution for specific isolates was obtained from the EUCAST database, and so may not necessarily reflect local susceptibility and resistance patterns. Importantly, urinary creatinine clearance was not directly measured in patients. Finally, no data on patient-centered outcomes such as clinical cure, ICU length of stay, or mortality were assessed.

### Conclusion

The most important highlight of this study is the large inter-individual PK variability, particularly in CSF, of PTZ in critically ill patients. No patient factors improved the ability of the population PK model to predict plasma and CSF piperacillin or tazobactam concentrations. High-dose piperacillin continuous infusions of 24 g over 24 h have the highest probability of attaining targeted plasma exposures, but therapy for CSF infections remains unsupported. Due to the small patient size and sparse CSF sampling, the PK model was not able to predict CSF piperacillin or tazobactam concentrations accurately enough to perform dosing simulations or make dosing recommendations to attain optimal CSF exposures. It remains unlikely that PTZ could be considered a therapeutic option for patients with VAI.
